# Demographic differences between health care workers who did or did not respond to a safety and organizational culture survey

**DOI:** 10.1186/1756-0500-4-328

**Published:** 2011-09-07

**Authors:** Tita A Listyowardojo, Raoul E Nap, Addie Johnson

**Affiliations:** 1 Faculty of Behavioral and Social Sciences, University of Groningen, Groningen, The Netherlands; 2 University Medical Center Groningen, Groningen, The Netherlands

## Abstract

**Background:**

Areas for institutional improvement to enhance patient safety are commonly identified by surveying health care workers' (HCWs) attitudes, values, beliefs, perceptions and assumptions regarding institutional practices. An ideal response rate of 100% is rarely achieved in such surveys, and non-response bias can occur when non-respondents differ from respondents on a dimension likely to influence survey conclusions. The conditions for non-response bias to occur can be detected by comparing demographic characteristics of respondents and non-respondents and relating any differences to findings in the literature of differences in the construct of interest as a function of these demographic characteristics. The current study takes this approach.

**Findings:**

All 5,609 HCWs at a university medical center were invited to participate in a survey measuring safety and organizational culture (response rate = 53.40%). Respondents indicated their professional group, gender, age group, years of working in the hospital and executive function. Because all HCWs were invited, the demographic composition of the group who did not respond was known. Differences in the demographic composition of respondents and non-respondents were compared using separate Pearson's chi-square tests for each demographic characteristic.

Nurses and clinical workers were generally more likely to respond than were physicians, laboratory workers and non-medical workers. Male HCWs were less likely to respond than were females, HCWs aged younger than 45 years old had a lower response rate than did HCWs aged 45 to 54 years old, HCWs who had worked in the hospital for less than 5 years were less likely to respond than were those who had worked in the hospital for 5 years or more and HCWs without an executive function were less likely to respond than were executives.

**Conclusions:**

Demographic characteristics can be linked to response rates and need to be considered in conducting surveys among HCWs. The possibility of non-response bias can be reduced by conducting analyses separately as a function of relevant demographic characteristics, sampling a higher percentage of groups that are known to be less likely to respond, or weighting responses with the reciprocal of the response rate for the respective demographic group.

## Background

Patient safety in the hospital depends on health care workers' (HCWs) commitment to safety (i.e., safety culture) [1-9]. Periodic assessment of safety culture is necessary in order to pinpoint attitudes, values, beliefs or perceptions that may need to be changed in order to improve patient safety [1,3-9]. Safety culture is typically assessed with surveys whose results are then used to design patient safety improvement programs, to evaluate the effectiveness of intervention programs and to track transformations of safety culture over time [1,3,4,8].

A challenge in surveying HCWs is to achieve a high response rate. Physicians, in particular, often show relatively low response rates [1,10-15]. For example, in a study by Singer et al. [1], the response rate of physicians was 33%, considerably lower than the response rate of 60% that is recommended for achieving sufficient reliability in the measurement of safety culture [5]. In fact, response rates of 60% or higher are rarely achieved [1,3]. A major problem with low response rates is that they may result in under- or over-representation of particular groups and, thus, in non-response bias [4,16-18]. Non-response bias is said to occur when a significant number of those who do not respond differ in terms of relevant characteristics (i.e., characteristics that can influence survey outcomes and conclusions) from those who do respond. Non-response bias is a real concern in health care settings. It has been found as a function of gender with regards to identifying best practices [19], duration of employment with regards to attitudes towards changes in national health programs [20], and age with regards to evaluation of alcohol abuse programs [21].

One way to detect non-response bias is to contact non-respondents personally in order to investigate whether non-respondents' opinions differ substantially from those of respondents [20,21]. This way of dealing with non-response cannot be applied when surveys are anonymous, as is likely to be the case when HCWs are surveyed [7,10,15]. However, when the demographic characteristics of the surveyed population are known, even anonymous surveys can benefit from comparing the demographic composition of respondents with that of non-respondents [12,16,19-22].

Although some safety culture surveys conducted in the past have considered the effects of demographic variables on how safety culture is evaluated [2,3,6,7,23], most studies of safety culture fail to consider the role of demographic characteristics in the evaluation of safety culture or make only vague reference to such differences [2]. An explanation for this might be that the goal of many studies of safety culture is to capture organizational (and not individual) factors that influence patient safety and that the importance of demographic differences in safety culture research has not been recognized [24]. We argue that demographic characteristics should be considered in understanding whether HCWs will be likely to respond to safety culture surveys and that they should be taken into account in reducing the risk of non-response bias for anonymously conducted surveys. The purpose of this study was thus to compare the demographic composition of the groups of HCWs who did or did not respond to a survey measuring safety and organizational culture. Differences in demographic characteristics of respondents and non-respondents that should be taken into account to reduce the risk of non-response bias are documented and suggestions are made for understanding the responding behavior and increasing the response rate of HCWs.

## Methods

### Participants

In April 2009, invitations to participate in a safety and organizational culture survey were sent electronically (via intranet) to 5,609 HCWs (out of a total of approximately 8,000 HCWs) involved in patient care (including managers) at the University Medical Center Groningen (UMCG), The Netherlands. The UMCG is a large university medical center that has approximately 1,300 beds, including 53 surgical and medical adult intensive care beds and 46 neonatal and pediatric intensive care beds. Reminders were sent in May and again in June. Participation was on a voluntary basis and no incentives to respond (financial or otherwise) were offered. Anonymity was ensured and informed consent was given by those who responded.

### Instrument

Nine dimensions of safety and organizational culture were assessed in a survey containing 99 items (see Additional File 1). Because the survey addressed general organizational concerns such as work satisfaction, working conditions and perceptions towards the hospital, in addition to aspects of safety culture, it was relevant to all HCWs who were invited to participate. Additional questions addressed department, gender, age, years of working in the hospital and whether one worked in an executive or non-executive function. Because some HCWs could belong to more than one department (e.g., a medical specialist who also teaches at the university belongs to both medical specialist and teaching departments), HCWs were asked to indicate the department where they spent most of their time as stated on their work contract. The ten departments were combined into five professional groups because of similar job descriptions (e.g., the medical specialist and physician assistant departments were combined). The five professional groups thus obtained were "physicians" (e.g., medical specialists and physician assistants), "nurses" (e.g., nurse practitioners and intensive care nurses), "clinical workers" (e.g., dieticians, psychologists and pharmacists), laboratory workers (e.g., laboratory technologists and technicians) and non-medical workers (e.g., facility management workers, secretarial and administrative workers and managers). The age groups used were 15-24 years old, 25-34 years old, 35-44 years old, 45-54 years old and older than 54 years old. The categories used for years of working in the hospital were less than 5 years, 5-9 years, 10-20 years and longer than 20 years. Members of any professional group were considered to hold an executive function if they led a sector, department, unit, subunit or clinic; data were coded according to the number of respondents who worked in an executive or non-executive function.

### Data analyses

Because of the need to preserve anonymity, data were available only for the demographic characteristics of the group and not for the conjunctions of the variables for each individual (i.e., we had available to us the data regarding how many, e.g., nurses responded, but not their age distribution, gender, etc., as this would uniquely identify individuals). It was therefore not possible to carry out multivariate analysis to identify which characteristics differed between groups of respondents and non-respondents while taking other characteristics into account. Instead, differences in the demographic characteristics of respondents and non-respondents were tested using Pearson's chi-square tests separately for each demographic characteristic (i.e., professional group, gender, age group, years of working in the hospital, and executive function), with group (respondents vs. non-respondents) and category (e.g., for the characteristic "gender" the categories were male and female) as factors. If interactions of group and category were found (i.e., if the demographic composition of respondents and non-respondents differed), follow-up pairwise Pearson's chi-square tests were conducted between sub-groups. Odds ratios (where an odds ratio of 1 indicates that the demographic composition of respondents and non-respondents did not differ) were used to calculate effect size. A significance level of *p *< .05, Bonferroni correction for each family of comparisons, was used where necessary.

## Results

Out of 5,609 invitations sent, 2,995 were responded to (response rate = 53.40%). Response rates as a function of demographic characteristic are summarized in Table 1.

**Table 1:** Response rates as a function of demographic characteristic

Category	Respondents (*n *= 2995*)*	Non-respondents (*n *= 2614*)*	Response rate (%)
Professional groups			
Physicians	496	570	46.53
Nurses	1208	871	58.10
Clinical workers	649	508	56.09
Laboratory worker	331	318	51.00
Non-medical workers	311	347	47.26

Gender			
Male	834	808	50.79
Female	2161	1806	54.47

Age group			
15-24 years old	122	156	43.88
25-34 years old	857	814	51.29
35-44 years old	735	664	52.54
45-54 years old	857	603	58.70
>54 years old	424	377	52.93

Years of working in the hospital			
<5 years	1059	1137	48.22
5-9 years	825	602	57.81
10-20 years	593	444	57.18
>20 years	518	431	54.58

Executive function			
Executive	167	75	69.01
Non-executive	2828	2539	52.69

Chi-square analyses (*n *= 5609) revealed interactions between group (respondents vs. non-respondents) and each of the demographic characteristics. The Group × Professional Group interaction (*X*^2^(4) = 53.54, *p *< .001) reflects that nurses and clinical workers had significantly higher response rates than did physicians and non-medical workers, and that nurses also had a significantly higher response rate than did laboratory workers (see Table 2 for all follow-up comparisons and odds ratios). The Group × Gender interaction (*X*^2 ^(1) = 6.33, *p *< .05) reflects that female HCWs had a higher response rate than did males. The Group × Age Group interaction (*X*^2 ^(4) = 30.07, *p *< .001) reflects that HCWs who were younger than 45 years old had significantly lower response rates than did those aged 45 to 54 years old. The Group × Years of Working in the Hospital interaction (*X*^2 ^(3) = 41.31, *p *< .001) reflects that HCWs who had worked in the hospital for less than five years had a significantly lower response rate than did those who had worked in the hospital for five years or more. The Group × Executive Function interaction (*X*^2 ^(1) = 24.77, *p *< .001) reflects that HCWs with an executive function had a significantly higher response rate than did those without an executive function.

**Table 2:** Chi-square results and odds ratios per sub-group

Category	*X*^2^	Odds Ratio	95% CI
**Professional group:**			
• Physicians vs. Nurses^1^	38.036**	1.59	1.37 - 1.85
• Physicians vs. Clinical workers^1^	20.319**	1.47	1.24 - 1.74
• Physicians vs. Laboratory workers	3.232	1.20	0.98 - 1.45
• Physicians vs. Non-medical workers	0.088	1.03	0.85 - 1.25
• Nurses vs. Clinical workers	1.23	0.92	0.80 - 1.07
• Nurses^1 ^vs. Laboratory workers	10.15*	0.75	0.63 - 0.90
• Nurses^1 ^vs. Non-medical workers	23.78**	0.65	0.54 - 0.77
• Clinical workers vs. Laboratory workers	4.343	0.81	0.67 - 0.99
• Clinical workers^1 ^vs. Non-medical workers	13.12**	0.70	0.58 - 0.85
• Laboratory workers vs. Non-medical workers	2	0.86	0.69 - 1.07

**Gender: **Male vs. female^1^	6.329	1.16	1.03 - 1.30

**Age group:**			
• 15-24 years old vs. 25-34 years old	5.223	1.35	1.04 - 1.74
• 15-24 years old vs. 35-44 years old	6.949	1.42	1.09 - 1.83
• 15-24 years old vs. 45-54 years old^1^	20.833**	1.82	1.40 - 2.35
• 15-24 years old vs. >54 years old	6.76	1.44	1.09 - 1.89
• 25-34 years old vs. 35-44 years old	0.477	1.05	0.91 - 1.21
• 25-34 years old vs. 45-54 years old^1^	17.278**	1.35	1.17 - 1.56
• 25-34 years old vs. >54 years old	0.588	1.07	0.90 - 1.26
• 35-44 years old vs. 45-54 years old^1^	10.99*	1.28	1.11 - 1.49
• 35-44 years old vs. >54 years old	0.032	1.02	0.85 - 1.21
• 45-54 years old vs. >54 years old	7	0.79	0.67 -0.94

**Years of working in the hospital:**			
• <5 years vs. 5-9 years^1^	31.867**	1.47	1.29 - 1.68
• <5 years vs. 10-20 years^1^	22.631**	1.43	1.24 - 1.66
• <5 years vs. >20 years^1^	10.721*	1.29	1.11 - 1.50
• 5-9 years vs. 10-20 years	0.097	0.97	0.83 - 1.15
• 5-9 years vs. >20 years	2.419	0.88	0.74 - 1.03
• 10-20 years vs. >20 years	1.36	0.90	0.75 - 1.07

**Executive function: **Executive^1 ^vs. Non-executive	24.771**	2.00	1.51 - 2.64

Bonferroni correction was used as appropriate for all analyses. ** *p *< .001. *p < .01.

^1^Had significantly higher response rate than the other sub-group.

## Discussion

The demographic composition of groups who did or did not respond to a survey of safety and organizational culture was analyzed and significant differences between groups of respondents and non-respondents were found. Response rate was found to depend on professional group, gender, age, years of working in the hospital and executive function.

The survey study on which the current study of demographic differences in response rates is based revealed not only differences in response rates, but differences in how aspects of safety and organizational culture are perceived [Unpublished data of T. A. Listyowardojo, R. E. Nap and A. Johnson]. The existence of differences in response rates as a function of demographic characteristics makes it important to consider whether non-response bias is likely to have influenced the interpretation of the survey results. In our study of safety and organizational culture, the data were analyzed and reported per group, and the major finding of the study was that perceptions of safety and organizational culture differed significantly across professional group, with physicians and non-medical workers tending to give more positive ratings of safety and organizational culture than did nurses, clinical workers and laboratory workers.

The key question addressed in this paper is whether non-response bias due to unequal representation of professional groups influences study results. If group composition is not taken into account when analyzing survey results, under- or over-representation of some groups can influence the conclusions that are drawn. For example, it might be that physicians and non-medical workers, who were relatively positive in their ratings of safety and organizational culture, are less likely to respond to safety culture surveys than are nurses and clinical workers because they feel that changes in institutional practices are not urgently needed. Basing conclusions on the response group as a whole could then lead to an overly negative evaluation of safety culture. More specific aspects of safety culture, such as fear of shame and blame [3], have been shown to be evaluated differently by nursing professionals than by physicians, and their evaluation may thus also be subject to non-response bias.

The relatively low response rate of physicians may also, in part, be due to the perception that they are too often asked to respond to surveys and that their time is too valuable to be spent completing them [12,20,25,26]. Whereas physicians have been found to complain about being asked to participate in surveys too often, nurses and clinical workers have reported that they are not asked for their professional views often enough [12]. Nurses and the clinical workers may thus embrace the opportunity to voice their points of view by responding to surveys.

The fact that gender affected response rate, with female HCWs being more likely to respond than their male colleagues may be tied to the fact that nurses are predominantly female, and nurses have a higher response rate. Because the effect of one demographic variable could not be statistically isolated from the others, we cannot say that females, in general, were more likely to respond.

The finding that HCWs in the 45 to 54 year old age group were more likely than were younger HCWs to participate contrasts with previous findings of higher response rates for younger HCWs [12,16,20,21]. The fact that these previous studies sampled only physicians (in contrast to the current study, in which all professional groups were surveyed) may be responsible for this difference in findings. Alternatively, it may be that HCWs in this age group were more likely to be senior staff with administrative duties.

The lower response rate of HCWs who had worked in the hospital for less than 5 years as compared to those who had worked for 5 years or more may be related to professional commitment to the hospital, which can be expected to increase as one works longer [25]. A final difference in response rates was that those who had an executive function were twice as likely to participate as were HCWs without an executive function. Those with executive functions may be more willing to participate because they are among those who will use the survey results to develop or defend patient safety intervention programs [6].

### Limitation

The main limitation of the current study is clearly the survey anonymity. The hospital would release only the demographic characteristics of the group as a whole and those of the group of respondents, and not those of the individuals. Furthermore, because we could not contact non-respondents, we were unable to investigate whether there was a difference in the evaluation of safety and organizational culture between respondents and non-respondents. However, previous studies have reported a strong link between relevant demographic characteristics (i.e., professional groups, age, years of work experience and executive function) and safety attitudes [1-3,6,24,27,28], making it possible to draw tentative conclusions about the possibility of non-response bias.

## Conclusion

Demographic characteristics can be linked to response rates and thus need to be taken into account in conducting surveys among HCWs. The possibility of non-response bias can be reduced by conducting analyses separately as a function of relevant demographic characteristics or by sampling a higher percentage of members of groups that are known to be less likely to respond [1-3]. Another approach to reducing the possibility of non-response bias is to weight responses with the reciprocal of the response rate for the respective demographic group [1,29].

## Competing interests

The authors declare that they have no competing interests.

## Authors' contributions

TAL contributed to study conception and design, data analysis and interpretation, drafting and critically revising the manuscript. REN contributed to study conception and design and acquisition of data. AJ contributed to data analysis and interpretation, and drafting and critically revising the manuscript. All authors read and approved the final manuscript.

## Supplementary Material

Additional file 1 **Overview of safety and organizational culture dimensions and sample items**. Overview of the dimensions included in the safety and organizational survey and sample items. Click here for file
