# Supplementary material for: Demographic differences between health care workers who did or did not respond to a safety and organizational culture survey
Source: BMC Res Notes. 2011 Sep 7;4:328. doi: 10.1186/1756-0500-4-328 (PMC3180706; doi:10.1186/1756-0500-4-328)
Supplement: Additional file 1 — Overview of safety and organizational culture dimensions and sample items. Overview of the dimensions included in the safety and organizational survey and sample items. [file 1756-0500-4-328-S1.PDF]

Additional file

Additional file 1 – Overview of safety and organizational culture dimensions and sample items

Dimensions	Sample items
**Institutional commitment to safety (8 items)**: perceptions of a strong and proactive organizational commitment to safety.	"I would feel safe being treated here as a patient", "The culture in my department makes it easy to learn from the errors of others".
**Teamwork climate (6 items)**: perceived quality of collaboration between hospital workers in delivering patient care.	"Disagreements in the department here are resolved appropriately (i.e., not *who* is right but *what* is best for the patient)", "It is easy for personnel in this department to ask questions when there is something that they do not understand".
**Team performance (12 items)**: perceived quality of team performance.	"My team delivers high quality results", "I experience good teamwork between different teams".
**Work satisfaction (16 items)**: positivity about work experiences.	"I enjoy my work", "I have sufficient opportunities to carry out tasks that I am good at doing".
**Working conditions (10 items)**: perceived quality of work environment and logistical support (e.g., schedules, workload, and equipment).	"I am satisfied with my working hours", "I am equipped with appropriate instruments and materials to carry out my work".
**Collegiality (8 items):** perceived quality of collegial atmosphere.	"I have pleasant colleagues", "I am motivated by my colleagues".
**Relations with supervisors (13 items)**: perceived quality of supervision.	"My supervisors give clear objectives", "I receive sufficient feedback from my supervisors".
**Perceptions towards the hospital (17 items)**: perceptions towards the organization's objectives, structures, and practices.	"I am proud of the hospital", "I support the objectives of the hospital".
**Career perspectives (9 items)**: perceived quality of self-development and career perspectives.	"At work, I can gain new knowledge sufficiently", "I have opportunities to develop my career in this hospital".
